# Infodemic: Challenges and solutions in topic discovery and data process

**DOI:** 10.1186/s13690-023-01179-z

**Published:** 2023-09-07

**Authors:** Jinjin Zhang, Yang Pan, Han Lin, Zhoubao Sun, Pingping Wu, Juan Tu

**Affiliations:** 1 School of Computer Science, Nanjing Audit University, Nanjing, China; 2 School of Engineering Audit, Jiangsu Key Laboratory of Public Project Audit, Nanjing Audit University, Nanjing, China; 3 The Institute of Acoustics, School of Physics, Nanjing University, Nanjing, China

**Keywords:** Infodemic, Data science, Review, Research topics, Communication

## Abstract

**Background:**

The Coronavirus Disease 2019 (COVID-19) pandemic was a huge shock to society, and the ensuing information problems had a huge impact on society at the same time. The urgent need to understand the Infodemic, i.e., the importance of the spread of false information related to the epidemic, has been highlighted. However, while there is a growing interest in this phenomenon, studies on the topic discovery, data collection, and data preparation phases of the information analysis process have been lacking.

**Objective:**

Since the epidemic is unprecedented and has not ended to this day, we aimed to examine the existing Infodemic-related literature from January 2019 to December 2022.

**Methods:**

We have systematically searched ScienceDirect and IEEE Xplore databases with some search limitations. From the searched literature we selected titles, abstracts and keywords, and limitations sections. We conducted an extensive structured literature search and analysis by filtering the literature and sorting out the available information.

**Results:**

A total of 47 papers ended up meeting the requirements of this review. Researchers in all of these literatures encountered different challenges, most of which were focused on the data collection step, with few challenges encountered in the data preparation phase and almost none in the topic discovery section. The challenges were mainly divided into the points of how to collect data quickly, how to get the required data samples, how to filter the data, what to do if the data set is too small, how to pick the right classifier and how to deal with topic drift and diversity. In addition, researchers have proposed partial solutions to the challenges, and we have also proposed possible solutions.

**Conclusions:**

This review found that Infodemic is a rapidly growing research area that attracts the interest of researchers from different disciplines. The number of studies in this field has increased significantly in recent years, with researchers from different countries, including the United States, India, and China. Infodemic topic discovery, data collection, and data preparation are not easy, and each step faces different challenges. While there is some research in this emerging field, there are still many challenges that need to be addressed. These findings highlight the need for more articles to address these issues and fill these gaps.

**Table:** 

Text box 1. Contributions to literature
• While Infodemic is now a hot topic, researchers are encountering various difficulties in its specific research topics and data analysis methods.
• We conducted an extended and structured literature analysis through which we identified challenges and proposed solutions in topic discovery and data process of Infodemic.
• Our findings are used to extend an existing framework on Infodemic analytics and provide benefits for researchers and practitioners who are interested in Infodemic.

## Introduction

In March 2020, the World Health Organization (WHO) declared COVID-19 a global pandemic. Subsequently, the organization cautioned that the novel coronavirus may remain a persistent threat, surpassing even terrorism. The outbreak’s impact has been unparalleled, and individuals require dependable and accurate information to make informed decisions. However, numerous false statements have been disseminated, leading to the creation of an “Infodemic”. The term Infodemic was first coined by Rothkopf in 2003 in his article “When the Buzz Bites Back.” He defined Infodemic as the dissemination of a few facts mixed with fear, speculation, and rumors, amplified and rapidly transmitted worldwide by modern information technologies. These actions have had disproportionate effects on national and international economies, politics, and security. In other words, information dissemination includes facts as well as rumors, misinformation and disinformation that may have a negative impact, which are not the same. Rumors refer to information that is unverified and unknown to be true or false; misinformation is that which is different from the facts due to negligence or unintentionally, without the intention to deceive; and disinformation is that which is deliberately generated and does not match the facts, with the intention to deceive and mislead, and is the most dangerous [1]. But they are all inconsistent with the facts and belong to false information.

False information about COVID-19 was widely circulated among the public and even promoted by the media or prominent individuals, and conspiracy theories about the vaccine even emerged at a later stage [2–4]. Some false information about COVID-19 is documented in Table 1.

**Table 1:** Different types of false information during the epidemic

Category	Definition	Example
Misinformation	Inadvertent or unintentional factual inaccuracies without intent to deceive.	“The novel coronavirus vaccine only protects for 6 months.”
Disinformation	Deliberately created factually incorrect information with the intent to deceive and mislead.	“The novel coronavirus may cause cancer.”
Rumor	Unverified information of unknown authenticity.	“A person can be infected with the novel coronavirus up to 8 times.”

Today, with the rapid development of science and technology, information spreads faster and more widely than viruses. We are not only fighting against viruses, but also fighting against Infodemic, Infodemic has also become a threat. If people believe false information, more than scientific guidelines, this can lead to a great danger to public health safety [5, 6]. Accuracy is only one of the things people care about when it comes to spreading information, and information spread by highly influential users is revered [7, 8]. Such widespread dissemination of false information has been seen in other infectious diseases, such as Ebola [9], yellow fever [10], has had a significant negative impact. And the negative impact of false information in the Infodemic is twofold. On the one hand, people’s distrust of vaccines and contempt for COVID-19 may lead to a reduction in the level of prevention. On the other hand, people are afraid of the impact of COVID-19, which will make people overestimate its impact and cause tension among the masses, and people may believe some rumors and lead to the wrong way of treatment [11].

The plethora of information and the difficulty of distinguishing between true and false makes it difficult for people to find reliable sources and guidance when they need it [12]. This fear of not being able to obtain the true information or not being able to distinguish between the true and false information makes the hidden information disease appear, which also leads to COVID-19 being taken seriously at an early stage of the epidemic. Repeated and detailed content about viruses, geographic statistics, and multiple sources of information can each lead to chronic stress and confusion in times of crisis. In addition, a large number of false information, rumors, and conspiracy theories spread every day [13]. Sylvie Briand, director of infectious disease hazard management for WHO’s health emergency program staff and architect of WHO’s strategy to address infectious disease risks, told *The Lancet*, “We know that every eruption is accompanied by a kind of information tsunami, but in that information, you also always have misinformation, rumors, and so on” [14].

In the age of epidemics, as epidemics continue to grow, so do infectious disease data. From early 2020 through the second half of 2021, Google Fact Checker recorded more than 7,000 false, misleading, and unsupported representations related to COVID-19, according to the survey [15]. In this context, how to combat false information in Infodemic has become a major hot topic. Infodemic data broadly includes text data, videos, images, audio, and so on. The growth of Infodemic data has driven the exchange of analysis in related fields. For example, Infodemic data is analyzed to see how rumors are generated [16] or to study models of how to combat epidemics [17], and so on. Infodemic, although proposed a long time ago, has only really become known to the public since COVID-19. While a growing number of relevant studies have arisen, most of the current relevant studies are isolated case studies. Researchers collect and analyze data on a specific topic over a certain period. They differ in what they study, but the research steps are essentially the same and the challenges faced in the study are usually similar. Before examining the data, researchers need to discover the topic, collect the data, and prepare the data.

Therefore, we conducted a systematic literature review, and we believe that the complexity of the equally important steps of topic discovery, data collection, and data preparation has not been fully covered in the study, and there is no uniform standard for how each step should be handled. In research, we only focus on the challenges or difficulties encountered by researchers in discovering topics, collecting data, and preparing data, rather than focusing on the purposes and methods of their analysis.

Our paper focuses on the following research question:

### Research question

What challenges do researchers face when it comes to topic discovery, data collection and preparation for further analysis of Infodemic?

The answer to this question can give future researchers ideas to prevent them from being overwhelmed when they are also faced with these challenges, and they can learn about the problems they will face before they acquire the data, rather than discovering them during the research process. At the same time, the answer is not useless to those who have already researched, it can help to give some insight into existing research, which will help to identify areas that may need further investigation or challenges that have not yet been adequately addressed. The contribution of this study is to summarize and provide some challenges in topic discovery, data collection and data preparation through a specific overview of related papers, and to suggest possible solutions and future research directions based on these challenges so that future researchers can take these difficulties into account in advance.

The remainder of our paper is broadly divided into the following sections: First, we provide the current state of research on the Infodemic related literature and the theoretical background of this paper. Second, we describe our research methodology and summarize the challenges and solutions we found based on our findings. At last, we summarize the whole paper, pointing out the limitations and suggesting possible future research directions.

## Theoretical background

The researchers divided the process of analyzing the Infodemic into several steps. We used three steps: topic discovery, data collection, and data preparation. However, the existing literature does not contain a comprehensive description to address the challenges in these steps.

One reason for the popularity of Infodemic is that people can obtain this information without paying a high cost, and this information is ubiquitous. In a crisis, if you do not have a certain source of information, it is easy to trust the words on the Internet or others, and it is difficult to tell whether these words are true. When everyone is in a panic, information comes out one after another. Some media will release a message in a hurry to keep up with current events without verification, and it is difficult to distinguish the truth from the false. Because of the complexity of these messages, analyzing Infodemic is a complex process, involving various fields and using different methods. Therefore, it is necessary to consider each step in advance and standardize it into a model.

Few research articles consider the issue of steps of Infodemic analysis. Chan et al. used the steps of “measure”, “extract”, and “analyze” in their Infodemic analysis [18]. And the authors studied this direction is the embodiment of the topic discovery. The authors pointed out that when measuring events, the data should be grouped and the range of data extracted should be determined. Then, irrelevant information, such as links, numbers, and user tags, needs to be removed during data analysis. Then, it is analyzed using data visualization analysis. Finally, statistical analysis is carried out. But the authors do not propose a framework to use. The study confirms that the use of social media for information research is an important area of development in public health. COVID-19 tweets are mainly used to disseminate information from reliable sources, but they are also sources of views, emotions and experiences [19]. Banerjee and Meena suggested how the role of social media (and other digital platforms) can be integrated into society and public health to achieve better symbiosis, “digital balance” and respond to the current crisis and prepare for future pandemics [13]. Stieglitz and Dang-Xuan have proposed a framework for social media analytics (SMA), which outlines SMA in three steps, including capture, understand, and present. The author noted that the framework was originally developed in the context of political communication [20]. In principle, it could easily be applied to other areas of research. The “capture” step in the framework includes data collection and data preprocessing, in which the required information is extracted and invalid information is eliminated. Therefore, these studies are different in the field of study or the objective of the analysis, but they are similar in the process. To sum up, we made some adjustments based on the framework based on your research and added the step of discovery topic to form the framework of Infodemic analysis. The SMA framework does not have this step because it does not require topic discovery. But when we do research, many themes in the Infodemic are unknown, topics that have not been discovered before and must be discovered.

Taken together, we propose the following four steps: topic discovery, data collection, data preparation, and data analysis. Topic discovery refers to the determination of research topics or fields, research objectives and directions. This will clarify the research objectives for the researchers and guide the subsequent stages such as data collection. Data collection refers to the process of obtaining data related to the topic through some technical methods. In this process, researchers need to determine data sources and collection methods to provide necessary information for subsequent stages of data preparation and analysis. The data preparation stage is to preprocess the collected data, which usually includes data cleaning, data integration, transformation, etc., so that high-quality data can be obtained and the subsequent data analysis process can be performed smoothly. Data analysis is the process of statistical analysis, visualization, and modeling of all the obtained pre-processed data to obtain relevant information about research issues.

## Methods

We will conduct a literature review to address the issues raised. The review can provide comprehensive information on the basis of the existing literature, so that later researchers or readers can spend less time to have a comprehensive understanding of this direction and provide a reference system for their research topics. “They set the goal to discourage researchers from using the same old theories and methods in a recycled and replete way” [21]. Among them, the process of literature retrieval plays an important role in the literature review. The quality of literature depends on the process of literature retrieval [22].

Therefore, our retrieval is roughly divided into the following steps. First, we searched for relevant studies in a specific database using a specific keyword. The second step is to classify the steps in Infodemic research based on the theoretical basis described above. In the cases we studied, there were different categories. The third step is to carefully examine the literature and see if there are any similarities and differences between the literature. It gives us a sense of the challenges researchers faced and the solutions they proposed to address them. This step can be a systematic integration of previous studies.

### Search methods

The purpose of this review is to examine the challenges of discovering a topic, collecting data, and preparing data for the Infodemic without considering the data analysis and subsequent results. For the literature search, we selected two major databases, including ScienceDirect and IEEE Xplore. ScienceDirect and IEEE Xplore cover a wide range of subject areas, including natural sciences, computer sciences, etc., which are a better match for the Infodemic of this study, and the quality and credibility of the literature are high. After comprehensive consideration, we chose these two databases that are more comprehensive relative to this study. Our main purpose was to study the challenges of finding a topic, gathering data, and preparing data for Infodemic, but Infodemic is a relatively new field and there is not much research on it. A search for the “Infodemic challenge” would yield very little literature. For example, the research on how to combat Infodemic rumors does not mention challenges, but it also goes through the discovery-collection-preparation-analysis phase, where there may be some difficulties [23]. Therefore, we used only one term for our search: “Infodemic”. However, because the search terms are not specific enough, the search results may result in a large amount of data. The stage of “topic discovery, data collection, data preparation and data analysis” is basically used for researchers conducting research on Infodemic data. Therefore, we limited the search topics of the ScienceDirect database to social science and computer science. Also, we limited the search scope to titles, summaries, and keywords. When searching, we referred to the list of references in the article to search for literature that was missed or not recognized during the search. Because Infodemic is a relatively new field and has only been researched in recent years, so we set the time frame for the search at January 2019 to December 2022.

### The inspection processes

To ensure that the literature was consistent with the topic of this study, the selected literature was assessed and screened for validity. We developed the following selection criteria to determine the relevance of the literature:

• Topic relevance: To determine whether the literature and our research topic are relevant, we first used semantic extraction and short text similarity techniques. The former helped us extract keyword information from the literature and was used to determine whether it was relevant to the topic. The latter was used to compare the degree of similarity between documents with insufficiently explicit keywords and texts that have been determined to be relevant. In addition, since our field is an emerging one and most of the challenges are mentioned only in the limitations section, we used keyword matching techniques to look for sections of the text with words such as “limitations” and “challenges”. Although natural language processing techniques can help to speed up the process of literature screening, they need to be combined with manual reading methods to ensure accuracy.

• Type of study: We prioritized the literature that used an empirical research design in order to facilitate the reliability of our study. Some of the literature studied issues related to Infodemic, but did not mention anything related to topic discovery, data collection, data preparation and data analysis phases, but only discussed Infodemic, such as the definition, causes and effects of Infodemic [24–27].

• Literature quality: we assessed the quality and credibility of each piece of literature. We focused on factors such as the level of detail of the study methods provided in the literature, the reasonableness of the sample size, and the appropriateness of the data analysis methods to ensure that the cited studies were highly reliable and relevant.

**Fig. 1:**
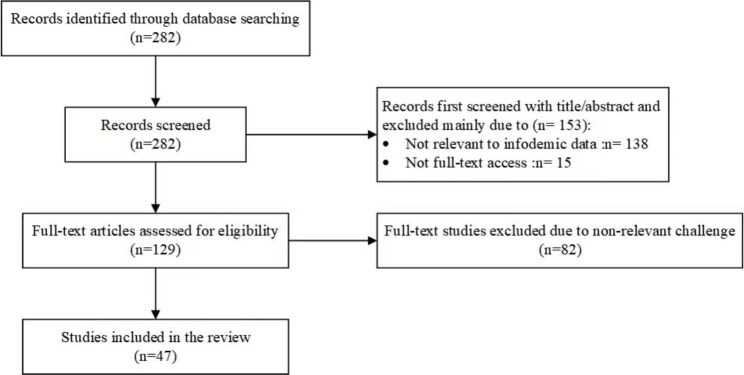
Literature screening process (January 2019 - December 2022)

Through these selection criteria, we excluded literature that was not relevant to the study topic and selected high-quality literature that met the study requirements. Figure 1 illustrates the screening process.

### Classification

We classified and labeled the 47 papers we finally determined according to topic discovery, data collection and data preparation stage. In addition, we added 3 topic discovery related literatures for complementary purposes. Table 2 shows all the relevant literature retrieved and marks which part of the challenge the literature refers to.

**Table 2:** The classification of relevant papers found in the systematic literature review

Database	Article	Phase		
		Topic Discovery	Data Collection	Data Preparation
ScienceDirect	[69] [70] [71] [47] [34] [33] [72] [73] [74] [75] [76] [77] [78] [79] [80] [81] [82] [83] [84] [85] [86] [87] [88] [35] [89] [36] [90] [91] [92] [69] [93] [94] [28] [95] [96]		√	
ScienceDirect	[42] [97] [66] [38]		√	√
IEEE	[98] [37] [41] [99]		√	
IEEE	[53] [100] [29] [30]		√	√
IEEE	[54]	√		
ScienceDirect	[55]	√		
Other	[56]	√		

## Findings

We examined the literature, identified common challenges encountered by researchers, found corresponding solutions, and summarized them. In summary, most of the challenges were concentrated in the data collection step, with few challenges encountered in the data preparation phase and almost none in the topic discovery section. At last, based on our research, we put everything together. However, we believe that topic discovery is a point that should not be ignored. Therefore, we reviewed other relevant literature to summarize the challenges in topic discovery. Table 3 summarizes the challenges faced and the corresponding solutions.

**Table 3:** Summary of challenges and related solutions

Challenges	Solutions	methods
Dynamic data.	Collect data samples quickly.	Use different data collection methods or develop models, such as digital tracking and survey data, LITMUS and so on.
Small data volume.	Obtain the required data samples.	Choose a dataset that is publicly available on the platform itself, or use computer tools to capture representative data.
Data complexity.	Filter data.	Use tools such as TF-IDF and BOW to obtain word frequencies and delete redundant information.
Overfitting during experiments.	Enhance data.	Use EDA, ENDA, AEDA, and other methods to generate new data or learn amplification strategies.
Misclassification.	Choose the right classifier.	Choose different classifiers according to different features of the dataset, such as NB if the dataset is small, SGD if high sensitivity values are needed, LR if the category is clear, Inception-v3 if it is image processing, ResNet if it is a computer vision task, and so on, and optimize the existing models.
Topic drift and diversity.	Use appropriate identification methods	Find the source of topic drift, choose a suitable method for recognizing topic drift, or use a suitable topic extraction model.

### Collect data samples quickly

#### Challenges

Nowadays, in the era of big data, data is undoubtedly the most important, and data is needed first to study any problem. And with Infodemic mainly being an information problem brought about by COVID-19, the amount of data is undoubtedly huge. But quantitative and rapid data collection in the context of emerging infectious disease outbreaks is challenging when face-to-face contact due to an epidemic is limited and when data are changing dramatically from moment to moment [28]. Data collection typically presents two challenges, one is high waste and the other is inefficient. First, collecting new messages requires continuous, effective real-time monitoring. And it is difficult to perform online monitoring when much of the data is not publicly available. Second, the data acquired is cumbersome, and Infodemic has much data that may never have appeared before, and the monitoring tools used may classify information that was not previously available as an outlier [29].

#### Solutions

Being able to collect a sample of data quickly is a technical challenge. When acquiring data, it is possible to use different data collection methods or develop models. Battiston et al. used a variety of complementary online data collection methods, including digital tracking and survey data, to collect first wave information [28]. Of course, some of the problems are not just with the sample itself. Maakoul et al. had problems collecting data when Facebook restricted the data and enforced certain procedures to obtain authorization, but they were not able to obtain the data even though they met the conditions according to the procedures [30]. Finally, they proposed to get more data by using proprietary special software or by increasing the time spent collecting data.

To obtain near real-time data more quickly, Musaev et al. developed the landslide information system (LITMUS) tool, which is a system for obtaining high-quality data from social networks in roughly real time, and was originally applied to landslide events [31]. However, it can be extended to other events by searching with keywords such as “COVD19, Coronavirus Pandemic, COVID-19, 2019 ncov Corona Outbreak, coronavirus, Wuhan Virus " and so forth [32]. Pu et al. have proposed to use tools such as LITMUS that can get the information we want from the application programming interface (API) we need in a timely manner, and the discussion about the epidemic has changed as new topics have emerged [29]. For example, when new drugs and vaccines are being developed, or when wrong messages and false messages appear in the system. Although new keywords can be added manually, the critical period for new discoveries may have passed, and the new information will disappear. So, this model can also be used to automatically add thematic keywords and track popular information by using social networks.

In conclusion, to collect data samples quickly, we need to rely on certain tools. Researchers can use already developed models or improve on them, mainly to be able to collect quickly and flexibly online.

### Obtain the required data samples

#### Challenges

Obtaining the necessary data is the top priority of the research. However, the nature of Infodemic makes it difficult for researchers to obtain data sets. The data set is small, not comprehensive enough, and the data collection relies on only a few specific populations with access to a specific platform [33, 34], which skews the results. Many studies are based on populations in individual countries, and cultural differences can cause differences in results, and people may react differently to an outbreak based on local customs. For example, some surveys were conducted only in Cyprus, which limited the generality of the results [35]. Similarly, collecting samples from certain age groups can lead to problems. For example, some data collection was done only by students, who may have had different preferences for variables than others, which could skew the results [28, 36]. However, the data survey done now is basically based on the Internet, and people who do not often use the Internet, such as older people over 60 years old do not use smartphones, cannot be tested online to obtain their data, and the use of offline questionnaires and other ways to spend too much time and labor. While some websites have restrictions on accessing data sets, Facebook limits data collection and specifies specific procedures for obtaining authorization [30], Twitter limits the scope of research for false information and does not allow rapid retrieval of data [37]. Social media is also a complex issue to obtain demographic data behind specific data [38], which concerns people’s privacy. Nowadays, although the Internet is all real-name, user information is not publicly available. And when the user information we have access to is a false identity, it can also have an impact on the research results.

#### Solutions

From the research point of view, collecting all the data would have been impossible, and selecting a sample that represents all public opinions cannot be done either. Therefore, in order to solve the problem of limited samples, we try to choose representative ones when selecting samples. We can screen the sample in each culturally representative country, and although there is still some variability among different countries, by means of increasing the sample coverage, we can also reduce the rate of bias in the results and increase the universality of the results.

There are two ways to obtain datasets. The first one is to choose a dataset that is publicly available on the platform itself, such as the COVID-19 dataset that Twitter has opened for research purposes [37], or choose the ones that have been accessed by previous researchers. The second one is the use of computer tools to crawl the data. When choosing the website to obtain data, try to avoid niche platforms and try to choose social networking sites that cover a wide range of people, such as Weibo, Twitter, and Facebook. when facing website restrictions on data collection, no good methods have been proposed. In this case, the collected data may contain a small amount of wrong information. Maakoul et al. have proposed using alternative techniques to solve this problem, such as comment extractors, but the results were not so good [30]. By combining the data collection method with the automatic error information recognition method, the data set size can be increased. Burel et al. have proposed that in order to obtain people’s true characteristics, researchers have studied methods to automatically identify various social media demographics and user account characteristics, with varying degrees of success and accuracy [38]. For people’s privacy issues, all records can be kept confidential through questionnaires and other means to ensure that the privacy of the respondents is not disclosed. When research is conducted face-to-face, researchers have the responsibility to ensure the privacy of the person providing the data [39]. When accessing data on the web, it is also important to ensure that the privacy of others is not violated and approval is obtained. During the data collection process, participants should be informed of the potential sensitivity of the study.

In addition, the data collected by the above methods, despite having initial validation, may still have error information, and therefore, the validators need to maintain caution when performing data validation. To reduce the impact of misinformation, multiple parties should be ensured to verify the consistency of the data and use appropriate validation methods, such as cross-validation methods that can help evaluate the characteristics of different data subsets; data duplication validation methods that can reduce the impact of errors in individual data sources, etc. Most importantly, data validation should be an iterative process that requires continuous monitoring and error correction. Only by continuously comparing and calibrating data can the accuracy and credibility of data be improved and the presence of erroneous information be minimized. However, this is a time-consuming process, and Hang et al. had proposed that the machine learning-enhanced graph analytics (MEGA) model used automatic feature-based vertex embedding to process the data to calculate accurate Infodemic risk scores [40]. In short, we have to choose the appropriate method to validate the data.

### Filter data

#### Challenges

Generally speaking, the acquired datasets are massive, such as Tweets contains a lot of unstructured, short and chaotic data, and the multimedia content is getting more and more complex, and there are also unimportant words appearing in these data, so how to select the reliable and useful parts from the massive data is a challenge to be solved in the preparation work.

#### Solutions

Considering that most of the collected data are textual data, numerous redundant textual information will inevitably appear in the preprocessing part of the data. For such information, term frequency–inverse document frequency (TF-IDF) can be used to calculate the number of occurrences of a word in the dataset so as to remove it, but it does not consider the location and contextual semantics of the word occurrences, so it cannot distinguish synonyms. So, bag-of-words model (BOW) is also needed, which gives the frequency of occurrence by discriminating the similarity of words. Of course, for keywords like COVID-19, there is no deletion operation at the same time. Also to avoid the effect due to case, it is also necessary to convert all the obtained information to lower case at the beginning [41]. Of course, we can also filter out the required reliable databases by filtering domain names when obtaining data. Gallotti et al. proposed a three-level filtering method that greatly reduced the data range and increases the probability of obtaining valid data [32].

### Enhance data

#### Challenges

In the field of data mining technology, data is the most basic part. People can obtain the required data through computer technology, but some qualitative data required by neural learning is difficult to obtain and will consume a lot of time, which needs to be expanded and processed later [42]. Many current deep learning models are overly complex and are more prone to overfitting with the small amount of data that Infodemic can access.

#### Solutions

Many researchers have focused on the processing phase of data and proposed some solutions. Data augmentation is the process of modifying and adjusting the original data when there is a lack of massive data and then expanding it to the original data to get double the amount of data. Data augmentation mainly has supervised data augmentation and unsupervised data augmentation methods. Among them, supervised data expansion is divided into single-sample data expansion and multiple-sample data expansion methods. By increasing the number and diversity of training samples, it helps to improve the robustness and generalization ability of the model and reduce the risk of overfitting, but it also relies on the raw labeled data excessively, and the generated expanded samples may inherit these problems if the labeling of the original dataset is of low quality or has errors. In contrast, unsupervised data expansion is divided into two directions: generating new data and learning expansion strategies. This expansion method does not require labeling information and can directly transform and expand the original samples, reducing the need and cost of labeled data. However, because of this, the generated expanded samples usually do not maintain the same label information as the original samples.

In general, there are three basic method classifications for data enhancement based on practical problems. First is the paraphrase-based approach, which makes the content of the obtained utterances expand within a certain variance interval based on appropriate changes, but its changes are more limited [43]. The easy data augmentation (EDA) developed by Wei and Zou is also widely used [44]. Next are noise-based methods including swapping, deletion, insertion, and word replacement, one of the simpler techniques is an easier data augmentation (AEDA), studied by Karimi et al. [45]. Data augmentation is performed by inserting different punctuation marks in the text as it happens, which is easier to implement compared to EDA above. Chen and Zhang have proposed the easy numeric data augmentation (ENDA) method, which is superior to the AEDA method, although both perform data augmentation operations by adding a certain amount of noise, but the former provides a better booster [42]. The last one is a sampling-based approach, lending a trained learning model that can generate more diverse data by sampling completely new data rather than changing existing data [46]. Digital augmentation exploits data augmentation and improves the performance and robustness of purpose-built classification models as the amount of training data increases. The authors argue that ENDA can create new training samples while retaining the original labels of the augmented text.

### Choose the right classifier

#### Challenges

While preprocessing the data, the researchers used automated tools to classify the data, and although they tried to classify the text using optimal classifiers, some errors were inevitable. Different data have different characteristics and have different target problems to solve. And different classifiers have their own processing power and characteristics, which can lead to processing errors if an inappropriate classifier is chosen. For example, the model used to identify gender also fails to identify non-binary gender [38], logistic regression (LR) and decision tree classifiers are more sensitive to noisy data and usually tend to overfit; passive-aggressive classifiers are often less stable in accuracy when combined with higher-order n-grammar (n > 3) [47]. An unsuitable classifier may not take full advantage of the features and structure of the data, resulting in less accurate classification results.

#### Solutions

There are many ways to choose a classifier. If your training set is small, choose a high bias or low variance classifier, such as naive bayes (NB). It is better than a low offset multivariable classifier because the latter will have overfitting. But as the training set grew larger, low-offset multivariable approaches became asymptotic errors, because they would produce lower asymptotic errors, since a high-offset, asymptotic classifier would not be asymptotic enough to provide an accurate model. Different classifiers have different advantages, and the selection should be judged according to the characteristics of the dataset. If classification of various texts is required, one can choose the NB supervised algorithm, which uses the conditional probability theorem to find the class of new samples, but the sample size of a category is much larger than other categories, and the NB algorithm may appear to be biased towards the category that appears more frequently, resulting in poorer classification of a few categories [48]. If high sensitivity values are required in terms of feature scaling, stochastic gradient descent (SGD) can be chosen, which is based on the working principle of support vector machines (SVM) and logistic regression convex loss functions and is also preferred for large data since it requires only one example per iteration [49]. LR is the best choice when the class of the target is well-defined, and it is a statistical function for analyzing data using one or more variables to find the final result [50]. To classify various images, one can choose Inception-v3, a 48-layer convolutional neural network that has been trained on millions of photos and can be loaded in the ImageNet database [51]. If various types of computer vision tasks are to be performed, residual network (ResNet) can be chosen, which is a powerful backbone model that uses jump connections to transfer the output from one layer to another, greatly reducing the fading disappearance problem. Moreover, it simplifies training while still maintaining high accuracy [52]. Another solution is to use a model created by someone else and optimize it. Some researchers have proposed that the way to solve the classifier problem is to use the latest extension of the gender multimodal deep learning system (M3) model, which will lead to more accurate results [38, 53]. The problem with this approach, however, is that some models have not yet been exposed. Future researchers can also focus on improving algorithms to improve the accuracy of classifiers and classify Infodemic information from a variety of perspectives.

### Use appropriate identification methods

#### Challenges

Overall, the challenges of topic discovery focus on two aspects: topic drift and diversity. Firstly, topic change is a dynamic process, especially in the field of Infodemic, where new topics emerge all the time, and what was an old topic can be replaced, and thematic drift occurs, contrary to the intent of the topic. In the same way, as time changes, the results of a study may lose their usefulness to the original topic, which also leads to the problem of timeliness [54]. The second issue is that over the past few years, research in the field of infodemic has revealed a variety of themes related to COVID-19. Tsao et al. identified six topics, such as information epidemics, public attitudes, and mental health, in order to comprehensively understand the impact of the COVID-19 pandemic [55]. As well, Boon-Itt and Skunkan identified six topics in their study of identifying tweets about COVID-19, such as “public knowledge of COVID-19 in news reports” [56]. Diverse topics may lead to information overload and information dispersion problems, making it difficult to process and sift through large amounts of information.

#### Solutions

To solve the problem of thematic drift, the most crucial thing is how to find out what causes thematic drift. Park et al. found that the sharing of personal experience is also a common source of thematic drift [57]. Mullick et al. referred to statements that may lead to thematic drift as drifting sentences, and the subsequent ones as following sentences, and they proposed to categorize the subjective and factual nature of drifting and following sentences in order to identify what kind of statements can lead to thematic drift, which is crucial for understanding thematic drift [54]. At the same time, they developed an “intelligent chatbot” to recognize topic drift and determined whether a particular user has posted statements that lead to topic drift. And for topic diversity, Tsao et al. mentioned extending topic search using more comprehensive keyword combinations to identify the most relevant keywords with high details, which can help solve the problem of incomplete keyword lists [55]. Han et al. proposed to analyze the microblog text data using a topic extraction and classification model combining the latent Dirichlet allocation (LDA) model and the random forest (RF) algorithm to mine the themes and public sentiment related to the COVID-19 pandemic, and determine the themes in terms of both temporal trend and spatial distribution [58].

## Discussion

Infodemic is a rapidly growing research field that has attracted the interest of researchers from diverse disciplines. In recent years, the number of studies in this field has significantly increased, with researchers from various countries including the United States, India, and China. However, future studies must be conducted under different cultural backgrounds as the results obtained from a single cultural perspective can be too biased. The COVID-19 pandemic has been a major focus of research in the Infodemic field, and WHO in the infodemic management training organized by itself suggested that health workers need to identify and debunk misinformation in a timely manner, and that efforts should be made to develop high-quality, accessible health information in different digital formats, so research on infodemic is necessary, every relevant study has contributed to our understanding of this global crisis [59].

### Research contributions

This study makes significant contributions by providing a systematic overview and summarizing some of the challenges in topic discovery, data collection, and data preparation. Although there are some researches in this emerging field, there are still many challenges that need to be addressed. In selected papers that record the steps of topic discovery, data collection, and data preparation, these parts are usually not described in detail. The primary focus of these papers is on analyzing data based on a topic, which occupies the majority of the article. Our research shows that researchers have encountered various challenges in data collection and preparation, but they have not yet encountered significant challenges in terms of themes. However, it also highlights that Infodemic topic discovery, data collection, and data preparation are not easy, and each step comes with different challenges. These findings emphasize the need for more articles to solve these problems and fill these gaps.

**Fig. 2:**
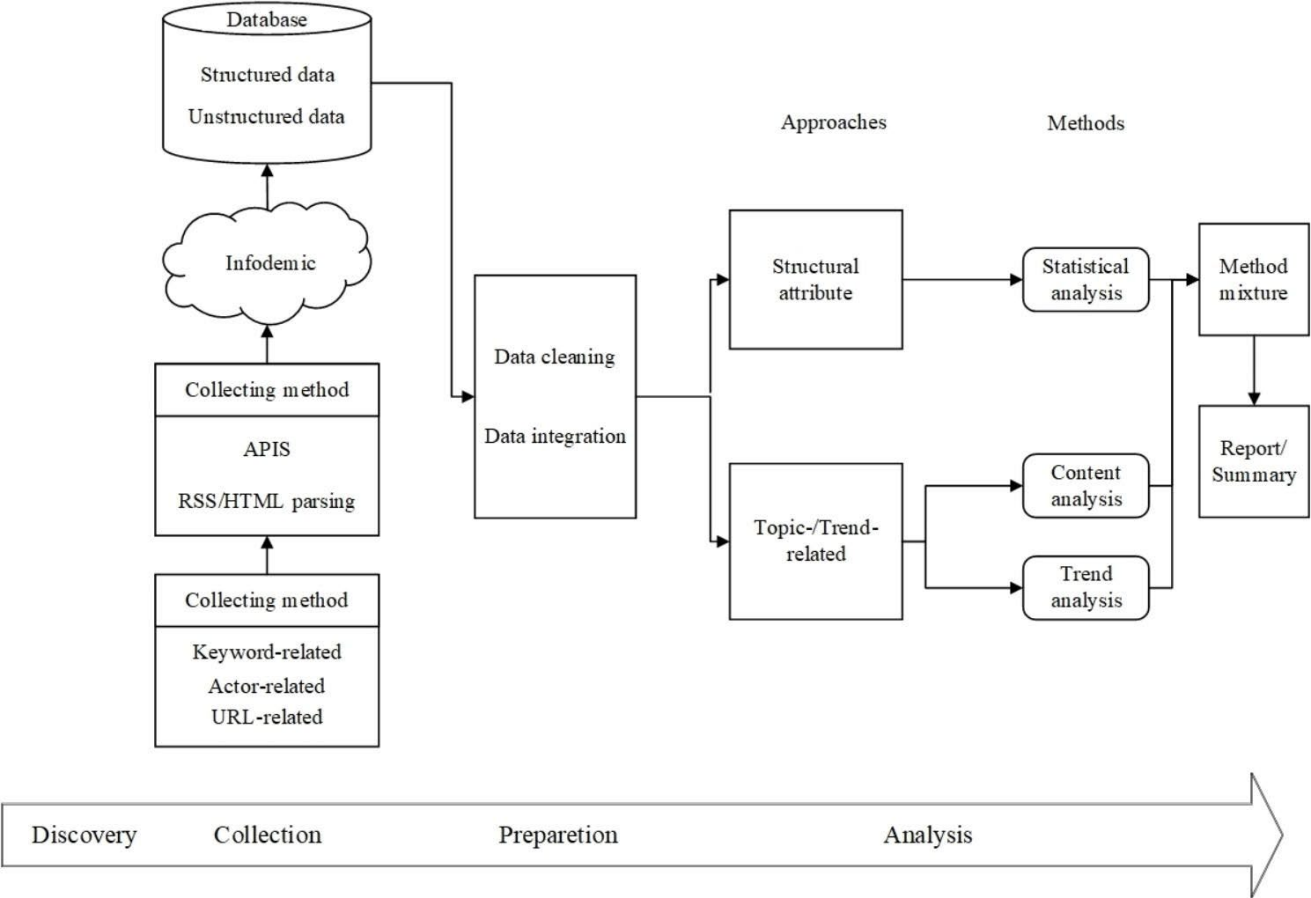
Infodemic Framework

To ensure the success of the project, researchers should plan everything in advance and take countermeasures before encountering challenges. We can assist in preparing in advance. As a second contribution, we suggest possible solutions to these challenges and future directions for researchers to consider. We examined some of the reviews, which basically centered on the causes and effects of Infodemic, with some limitations. Bella et al.’s article describes problems that arose in the early stages of the pandemic, which bringed up the issue of timeliness of the topic, and similarly, they elaborated on some issues related to limitations of the data sources [60]. Chowdhury et al. in order to have a wide range of research, fully collected the aspects of misinformation in the context of an outbreak, which leaded to a diversity of topics that may seem comprehensive, but in fact there were many redundancies [61]. Sasidharan et al. argued that if there is an overabundance of articles to justify rumors or not, which also leaded to an increase in information epidemics, then for this type of problem we should filter the data effectively [62]. Corinti et al.’s data includes only English language sources and does not fully cover a global sample of data, which requires more collection of the needed data [63]. Overall, this study provides a comprehensive analysis of the challenges in topic discovery, data collection, and data preparation in the emerging field of Infodemic. It highlights the need for further research and provides practical guidance for researchers to address these challenges. Based on the framework of SMA, we proposed a framework for Infodemic. In the discovery stage, the topic that can be experimented is discovered and proposed. In the collecting stage, the collecting process is described. Methods such as really simple syndication (RSS) or hypertext markup language (HTML) are used to search content related to uniform resource locator (URL), keywords and actors to obtain structured or unstructured data. In the preparation phase, the data is preprocessed, such as data cleaning, data integration, etc. In the analysis stage, the appropriate analysis method is selected, such as identifying structural attributes, themes, and trend related patterns, and then conducting statistical analysis, content analysis, or trend analysis. Figure 2 illustrates our findings visually.

### Limitations

However, this study has some limitations that need to be considered. Firstly, we used a specific database which, while representative, may have thematic omissions due to its one-sided nature. Secondly, we were unable to obtain permission to access some documents, which may have resulted in omission of important results. Finally, certain challenges were raised, but we were unable to find a solution, which is a subject for future research. Additionally, solutions proposed by some researchers may not be applicable to all studies.

### Future research directions

COVID-19 is unlikely to end soon [64], and the Infodemic is a growing problem. The mix of true and false information on the internet hinders people from obtaining accurate information about the spread, prevention, and treatment of the disease, leading to long-term information problems. The integration of informatics and epidemiology is crucial for future research to improve the global health security of people [65]. False information, whether propagated online or offline, can have negative impacts. The current situation highlights the urgent need to slow down or even stop the expansion of the Infodemic problem. Future research can focus on the following directions.

First, improving tools and theories to identify false information is crucial. Given the rapid changes in information, we need to monitor and identify online rumors in real-time, study their sources, purposes, and dissemination channels, and perform more thorough analysis of all kinds of information related to COVID-19 [37]. Despite the establishment of databases with sufficient information and development of tools to detect false information, more attention should be given to ontology-based analysis and the use of various targeted learning models to obtain more accurate results. The development of more accurate false information identification models will help people identify and reduce the spread of false information, thus providing a reliable information screening tool, reducing misinformation and panic, and increasing public trust, which will help increase confidence in public health communication.

Second, expanding the sample size acquisition is essential. Previous studies on the Infodemic have limited sample sizes, focusing on a specific social media platform or certain types of false information. Future studies should expand the research scope, increase the sample size, and select representative samples for experiments [24]. Researchers can also employ offline research methods such as face-to-face interviews to obtain valid data for false information that is different from online sources and may provide new insights. Expanding the sample size of a study provides more accurate information, supports more refined analysis, and the larger sample size reduces the effect of chance or error and the likelihood of researcher misclassification. In addition, large sample sizes help reveal more subtle effects and correlations in Infodemic, allowing researchers to develop more targeted methods. However, it is important not to blindly pursue large sample sizes at the expense of design rationality, sample quality, and methodological feasibility, and requires more consideration of other factors.

Third, enhancing the impact of reducing this factor of cognitive impairment is key. Infodemic affects people differently, and future research can investigate the factors that influence people’s refutation or acceptance of false information. Wang et al. proposed the moderating role of cognitive ability and found that by increasing cognitive ability, people’s ability to refute false information improves, mitigating the negative effects of false information [66]. However, different results have emerged in studies, such as the negative moderating effect of cognitive ability between argument quality and rebuttal acceptance. The contrasting results may be due to several factors, such as differences in study design, differences in sample characteristics, and differences in study methodology. Different researchers have different focuses, and future studies could adopt a more comprehensive approach that considers the effects of different factors to reveal the moderating effect of cognitive ability on people’s ability to accept or refute disinformation in order to cope with Infodemic.

Fourth, interdisciplinary field crossover is necessary. Since the outbreak of the COVID-19 epidemic, WHO has expanded the concept of information epidemiology to a multidisciplinary scientific field. Experts from various disciplines also have different views and different ideas about the discipline of information epidemiology [67]. How to deepen the integration between the discipline of information epidemiology and different disciplines is also something that needs to be studied by experts in the future. For example, the social field could draw on the knowledge of crisis managers in crisis management, while scholars in the computer field could collaborate with medical professionals to train more refined learning models to improve the accuracy of distinguishing rumors about various diseases. However, it should be noted that cross-disciplinary cooperation presupposes that scholars from various disciplines establish a good trusting and cooperative relationship, because effective trust building is important for both academic research and real-world applications [68].

## Conclusion

This review describes the current status of research, theoretical background, and research process, summarizes the challenges encountered by researchers studying Infodemic related experiments in terms of topic discovery, data collection, and data preparation, and proposes corresponding solutions. At present, it seems that the challenges in topic discovery are almost absent because of the emerging field, but it does not mean that future researchers will not encounter related problems, while there are many challenges and difficulties in data collection and preparation. Therefore, researchers should consider the following questions before starting their research: How quickly can we get data? What is the expected amount of data? How do we select a representative sample of data? Which classifier should we choose to process the data? Do we have enough infrastructure to handle the data when we collect and prepare it? This article can help researchers obtain answers in advance, enabling them to proceed more smoothly to the next step in their research.

## Data Availability

Data involved in this study is from ScienceDirect and IEEE Xplore databases.
